# Exploring the patient’s recovery journey and information needs following a shoulder fracture: A qualitative interview study

**DOI:** 10.1371/journal.pone.0316516

**Published:** 2024-12-31

**Authors:** Pauline May, Firoza Davies, Gillian Yeowell, Chris Littlewood

**Affiliations:** 1 Integrated Musculoskeletal, Pain and Rheumatology Service (IMPReS), East Lancashire Hospitals NHS Trust, Burnley, United Kingdom; 2 Faculty of Health, Social Care and Medicine, Edge Hill University, Ormskirk, United Kingdom; 3 Patient Representative, East Lancashire Hospitals NHS Trust, Burnley, United Kingdom; 4 Department of Health Professions, Manchester Metropolitan University, Manchester, United Kingdom; 5 School of Health & Society, University of Salford, Salford, United Kingdom; Northumbria University, UNITED KINGDOM OF GREAT BRITAIN AND NORTHERN IRELAND

## Abstract

**Background:**

Shoulder fractures (proximal humerus fractures) are common, painful, debilitating injuries. Recovery is a long process often hindered by complications such as mal-union and frozen shoulder. The purpose of this qualitative study was to explore the experiences and information needs of people at different time points after a shoulder fracture and how views on recovery change over time.

**Methods:**

This longitudinal telephone interview study used a semi-structured approach based on a pre-planned interview topic guide. Recruitment was from June to November 2023. Participants were interviewed approximately two months and five to six months after their injury. Interviews were audio-recorded and transcribed verbatim. Data were analysed using thematic analysis.

**Results:**

14 participants were recruited (age range 44–80 years; three male). The themes identified were dependence, vulnerability, information needs, and recovery. Loss of function and identity were associated with dependence. Feelings of vulnerability were present for most participants at six months post-injury. Information needs evolved, with information about the extent of the injury and practical advice needed first, but later participants emphasized the importance of reassurance and expected timelines for recovery. Recovery meant regaining function and independence, and returning to meaningful activities, which was also not fully achieved for most participants by six months.

**Conclusions:**

This study is the first to explore information needs and experiences along the timeline of recovery from a shoulder fracture. What recovery means to individual patients, along with recognition of the extent to which feelings of vulnerability affect recovery are important factors to consider. Clinicians should be aware of the full impact of these injuries to guide patients on their recovery journey, including identifying feelings of vulnerability and regaining their identity. Adopting a person-centred care approach, and considering the changing priorities and information needs of patients throughout their recovery journey may lead to improved patient care.

## Introduction

Shoulder fractures (proximal humerus fractures) are common, painful injuries and incidence rates are rising in line with an ageing population [1]. These injuries are more common in women, with the typical mechanism of injury being a fall from a standing height [2]. Problems such as pain, lack of movement, and reductions in strength can last for many months, and some people do not get back to their previous levels of function [3–5]. Recovery can be hindered by complications such as mal-union and frozen shoulder [6], an increased risk of re-hospitalisation, or further fracture [7]. Previous studies have reported that pain, and loss of function and independence are common following fractures, including shoulder, ankle and wrist fractures [8–11]. Improving pain, restoring function and independence were what patients valued the most [8,9]. McKeown *et al*. [10] also reported on a study of people who had sustained an ankle fracture, stating that independence and function are important factors in recovery. People who have sustained a distal radius fracture (wrist fracture) have been reported to have concerns regarding dependency, pain, and fear, including fear of falling, fear of pain and fear for the future [11].

There has been no study to date that has looked at the patient experience over the timeline of their recovery following a shoulder fracture, and how this, and their information needs, might change. Appropriate information provided at the right time may lead to improved recovery for patients after a shoulder fracture and may help to empower patients to make informed decisions regarding their health care and to take a proactive approach in their recovery [12]. Recovery has been found to be both a process and an outcome for patients after musculoskeletal trauma [13]. Information leaflets relating to injury, rehabilitation and recovery that are given to people following this injury can be difficult for patients to understand [14]. To ensure that information is accessible and appropriate, it is necessary to understand the patients’ experience of living with a shoulder fracture and what is important to those who have sustained this injury. Thus, this study investigated the following research question: What is the experience of people living with a shoulder (proximal humerus) fracture and how does information provision support recovery for people who have sustained these injuries?

## Methods

This qualitative study is one step in a planned multi-methods programme of research that is presented within a pragmatic framework. Pragmatism is based on the belief that knowledge is directly linked to experience [15] so this programme of research fits well within this paradigm, as it is an exploration of the participants’ experiences. The initial stage of this concerned the analysis of the content of information sheets for patients following a shoulder fracture [14,16]. The next planned stage is the co-design and development of an appropriate information leaflet for this cohort of patients (see S2 File. Flow chart of programme of work). The study was registered with clinicaltrials.gov prior to commencement and is reported in accordance with the consolidated criteria for reporting qualitative research [COREQ] [17]. Ethical Approval was gained from the Proportionate Review Sub-committee of the East Midlands—Nottingham 1 Research Ethics Committee on 05 May 2023. REC reference: 23/EM/0115.

Adult patients referred to outpatient physiotherapy in one NHS Trust in Northwest England following a shoulder fracture were identified as eligible for inclusion (Table 1) and recruited between 14^th^ of June to 28^th^ of November 2023. Potential participants were provided with a participant information sheet by post, and this was followed up with a telephone call to discuss participation. Written informed consent was gained prior to undertaking the interviews. The recruitment target was up to 15 participants to enable sufficient information power to adequately address the study objectives. This number was decided on by discussion within the experienced qualitative research team taking into account the information power guide as described by Malterud *et al*. [18]. Sampling was purposive regarding age, gender, levels of deprivation (based on the index of multiple deprivation (IMD) linked to postcode) and ethnicity, to gain a diverse spread of viewpoints and experiences among participants.

**Table 1:** Inclusion and exclusion criteria.

Inclusion	Exclusion
Proximal humerus fracture of any type	Under 18 years of age
Referred to outpatient physiotherapy from fracture clinic	
Able to give written consent	

An interview topic guide was developed from a review of the literature, the expertise of the research team and with a Patient and Public Involvement (PPI) Group. The PPI group consisted of 5 former NHS patients, all of whom sustained a shoulder fracture. The group’s age range was 49–84 years old, three were female, four identified as White British and one as Indian, the IMD range was from one to nine, where one is living in an area in the 10% most deprived in England and nine is living in an area in the 10% least deprived. The PPI group were also consulted during protocol development and assisted with the development of the consent form, participant information sheet and interview topic guide. The PPI group were consulted in relation to theme generation and potential implications of the study findings.

Individual, semi-structured telephone interviews were carried out by the lead researcher (PM). PM is a female researcher, who is a physiotherapist by background (with over 10 years’ experience) and trained in qualitative research (with 2 years’ experience). The researcher’s positionality can influence the direction and outcomes of a research study [19,20]. The position of outsider is considered to be one who is outside the group they are studying, whereas, one who is part of the community being studied. [20]. PM, being unknown to the participants, and never having sustained a shoulder fracture, has an outsider positionality with respect to the participants in the study. However, she did have significant knowledge of the service to which the participants were referred, having worked in that service for over eight years. The wider research team has an outsider perspective, having no knowledge of the participants or the particular physiotherapy service, though two (GY and CL) are physiotherapists by training. Telephone interviews were considered to be the optimal choice, in order to reduce the travel burden on participants, as the catchment area for recruitment was large. Telephone technology was deemed to be accessible to the vast majority of potential participants, more so than video conferencing technology.

The timeframes of interview were decided from a pragmatic perspective with the first interviews being approximately two months after injury. This time delay was due to the average referral time from fracture clinic to physiotherapy. A second interview time of five to six months was considered a reasonable timeframe for some recovery to have been achieved but also by the time limitations of the study funding.

The data were analysed using thematic analysis according to the six-step approach as outlined by Braun and Clarke [21]. A semantic approach was used, in that the data was analysed as communicated by the participant and underlying hidden meanings were not explored [22]. The interviews were audio-recorded, pseudonymized and transcribed verbatim by PM, using the software package NVivo (Version 12 Plus). PM checked the transcripts for accuracy and re-read them for familiarity (stage one), after which the initial codes were generated by identifying text relevant to the research question (stage two). An initial list of 54 codes were generated. These codes were iteratively explored and then iteratively refined. Cognate codes were grouped, and sub-themes were developed (stage three). During critical discussion with the co-authors (CL and GY), the codes were reviewed and grouped into preliminary themes (stage four). The preliminary themes and subthemes were critically discussed and refines into themes (stage five). The final stage (stage six) involved reporting of the data analysis within this paper. A reflexive diary was used to record the interviewer’s thoughts, observations, beliefs and biases, which were explored throughout the study. Preliminary analysis was undertaken after each interview, which enabled an iterative process of subsequent data generation.

## Results

48 patients were identified as potentially eligible to participate in the study. 29 of these were purposively selected to gain a diverse spread of participants, and therefore, a more diverse spread of viewpoints, and sent a participant information sheet and consent form, and then contacted by telephone to discuss participation in the study. Six declined to participate and did not state a reason; seven were unable to be contacted.

Sixteen participants provided consent and 14 were interviewed (mean age 64; range 44–80 years; three male; 12 White British, one Indian, one Pakistani; IMD range one to nine) (Table 2). The reasons for not participating were: one person needed to cancel due to work commitments and was unable to be subsequently contacted; one person was unable to be contacted to arrange an interview. Fourteen participants were interviewed in the first round of interviews (mean time from injury was 7.8 weeks; range five to12 weeks). Sufficient data was achieved to deliver on the study aims after 14 interviews in the initial round, so recruitment was stopped at 14 participants. Eleven participants were interviewed in the second round of interviews (mean time from injury was 5.5 Months; range 4.5–6 Months). Participant interviews lasted a mean of 20 minutes (range 10–34) across both rounds.

**Table 2:** Table of characteristics.

Participant ID number ^a^	Age at time of injury (Years)	Sex	IMD ^b^	Ethnicity (self-reported)	Dominant/ non-dominant arm injured	Time from injury to first interview (weeks)	Time from injury to second interview (months)
Participant 1	44	F	2	WB	Non D	6	5.5
Participant 2	71	F	2	Indian	D	6	Declined—no reason
Participant 4	49	F	2	WB	Non D	8	6
Participant 5	69	F	5	WB	Non D	10	6
Participant 6	51	F	1	Pakistani	D	12	Declined—no reason
Participant 8	72	F	8	WB	Non D	5	5.5
Participant 9	71	F	3	WB	Non D	12	5.5
Participant 10	64	M	2	WB	D	8	5.5
Participant 11	77	F	6	WB	D	6	Declined—Bereavement
Participant 12	48	M	2	WB	D	10	5.5
Participant 13	56	F	4	WB	Non D	6	6
Participant 14	72	F	9	WB	Non D	7	5.5
Participant 15	80	M	7	WB	D	6	4.5
Participant 16	75	F	2	WB	D	8	6

^a^ Not consecutive as participant numbers were assigned to those who consented.

^b^ IMD (Index of Multiple Deprivation): 1 is living in an area of most deprivation, 10 is living in an area of least deprivation.

WB White British; F Female; M Male; D Dominant; Non D Non Dominant.

Four themes were identified which are summarised in Table 3. Anonymised participant quotes have been used to support the findings. Psuedonyms have been suffixed by A and B to correspond to the first and second interviews respectively e.g. a quote followed by Participant 5-B means it is taken from the second interview with participant 5.

**Table 3:** Summary of themes and subthemes.

Theme	Subtheme
Theme 1: “*I felt that I was too reliant on everybody*”: Patient dependence after a shoulder fracture	Loss of function Loss of independence Loss of identity Change in dependence over time
Theme 2: “*This experience has made me realise …*, *that I’m not invincible”*: Vulnerability after a shoulder fracture	Ageing Fear of falling Isolation Change in vulnerability over time
Theme 3: “*I said you better put it in proper language”*: Communication and information needs	Communication Abandonment Guidance Change in information needs over time
Theme 4:”*Getting back to*, *you know*, *as I were before my accident”*: Recovery after a shoulder fracture	Achieving recovery Factors that influence recovery Change in recovery over time

### Theme 1: “*I felt that I was too reliant on everybody*”: Patient dependence after a shoulder fracture

#### Loss of function

Pain and lack of movement significantly affected participants’ ability to perform functional tasks, especially in the early stages after their injury, when the pain was often reported as the most severe.

*“I just couldn’t move it*, *rotate it*, *lift my arm*. *It just felt like it was kind of stuck and sore*, *like bruised*. *It was really painful.”*

Participant 13-A

#### Loss of independence

This loss of function influenced how well the participants carried out basic self-care activities including washing and dressing, which led to a loss of independence. All participants felt dependent in some way on family and friends needing them to help with self-care activities. Some participants reported feeling frustrated by their lack of independence and as if they were a burden on their family.

*“I felt like I was too reliant on everybody*, *because my own family and my partner have got their own things to do and I felt like I was stopping them doing whatever they had to do.”*

Participant 4-B

*“She needed help with*, *like*, *showering herself*, *bathing herself*, *dressing*.*”*

Participant 2-A (via interpreter)

#### Loss of identity

Some participants reported feeling a change in identity, one needing parents to care for her and another needing her children to help her with personal care tasks.

*“For the first 2 or 3 weeks were horrendous*, *my Mum had to actually get me in the shower*, *wait while I was in the shower*, *get me out of the shower*, *dress me*. *It was like going back to being a child basically.”*

Participant 1-A

“*You don’t like to rely on your child to have to get you out of bed*, *clothe you*. *You know*, *take you to the toilet and shower*… *it’s your worstest nightmare*, *you know really*. *Your children*, *you know to actually*, *go through that*. *It’s horrible*. *You lose everything*. *Your children have to see things that*, *you know*, *that you don’t want them to see*. *It took a lot out of me*.”

Participant 6-A

Despite the significance of this early dependence, by the time of the second interview (4.5 to 6 months post injury), participants typically reported that their ability to do daily self-care activities had improved.

*“I can cook my own meals; I can have a wash*. *I can do a lot more lifting as well*. *I can lift my arm and everything and it’s not as sore.”*

Participant 4-B

#### Change in dependence over time

Some participants had returned to further activities such as driving, by the time of the second interview. However, this was not the case for all participants, who did not feel that they were ready to return to some more challenging activities like work and hobbies.

*“I couldn’t drive initially*, *so that took me about*, *I would say about four months before I could drive comfortably.”*

Participant 13-B

*“I did do ballet classes*, *adult ballet but em*, *I haven’t gone back to that*. *I’m going to start going back in the New Year with that one because that is obviously all arms and things*, *so I didn’t want to go back until I felt like I was ready for doing that*.*”*

Participant 8-B

*“I had five months off work and I’m now back at work but I’m still on the phased return*, *doing lighter duties*.*”*

Participant 13-B

### Theme 2: “*This experience has made me realise …*, *that I’m not invincible”*: Vulnerability after a shoulder fracture

#### Ageing

Most participants reported feelings of vulnerability, with some feeling that the injury had also aged them.

“But I’m not young anymore. And I’m feeling it. I’m feeling the ageing” 9-B

“*This experience has made me realise that I am*, *it’s been a rude awakening*, *you know*, *that I’m not invincible [laughing]*”

Participant 14-A

#### Fear of falling

Many reported a fear of falling and reduced confidence when they were out walking.

“*I’m having to use a walking stick ‘cause I wasn’t unsteady before I broke my shoulder*. *It’s like I’m*, *I’m frightened of falling all the time*.”

Participant 15-A

#### Isolation

This vulnerability and fear of falling culminated for some participants in a lack of confidence in their ability to go out into the community. Three participants reported a sense of isolation as they felt like they couldn’t go out.

*“At the beginning I didn’t want anybody near me*. *I didn’t want to go out*, *well you couldn’t go out*, *could I really? I was bound to home.”*

Participant 6-A

#### Change in vulnerability over time

Some participants reported feeling that although they had been vulnerable initially, their confidence has been returning.

“*I mean I am worried about falling more definitely*, *because you know that that’s what could do the damage but em*, *no*, *my confidence is coming back*, *slowly*. *The more I can do*, *which is getting better every day*, *the more confident I’m getting*.”

Participant 1-A

### Theme 3: “*I said you better put it in proper language”*: Communication and information needs

#### Communication

The high levels of pain associated with the shoulder fracture affected some participants’ ability to absorb information during their clinical consultation, and one was glad to have family members present who could also listen to the information given.

*“I was in so much pain I couldn’t have taken in any more information anyway.”*

Participant 5-A

“*My daughter were with me or my husband were with me*… *So*, *they said*, *look Mum*, *the doctor said this*. *Because at that time I probably wasn’t even you know*, *taking in what they were saying at that time*.”

Participant 6-A

There was uncertainty around the extent of the injury (some people would have liked to be shown their x-ray) and the timeline to recovery.

“*It would have been nice if I got more… it’s difficult for me to interpret eh the fracture*, *you know*. *If I could have been shown the x-ray*, *‘cause that would have give me*, *you know more of an indication of the extent of the injury*.”

Participant 10-A

Some participants felt that there wasn’t enough time to discuss the injury in detail and ask questions.

“*I’ve had two consultation appointments*. *They were very brief to be honest*. *I didn’t feel like any time was really taken*, *…*, *the consultant saw me for about two minutes on my last appointment*.”

Participant 1-B

Due to the lack of information provided during the initial clinical consultations, some participants looked online for further information.

“*And of course*, *I’ve been on the internet*, *and I’ve been looking at what it says on the internet about when your humerus bone is broken*.”

Participant 14-A

Some participants reported the use of complex language in consultations or in clinic letters. Some felt able to ask the clinicians for more information.

“*Well*, *the thing is*, *they come out with these long words*, *and I said you better put it in proper language*, *you know [laughing]*. *But they were very kind*, *they explained everything*.”

Participant 16-A

“*The injury is closed*, *and she remains distally neurovascularly intact*. *I don’t really know what that means*.”

Participant 14-A (reading from the clinic letter during the interview)

Some participants reported good experiences regarding information and communication.

“*He said to me*, *this part of your shoulder that’s fractured*, *and it’s fractured lengthways not crossways so you’re not going to need surgery*, *but it is going to take some time to heal*, *and he was quite informative actually*.”

Participant 13-A

#### Abandonment

Many participants reported that they felt that there was a long wait for their first clinical consultation, and that they needed more guidance in the early stages after their injury. They reported a lack of clear direction in the initial stages of the recovery process which led to a sense of abandonment.

“*Eh*, *but I couldn’t get an appointment for about two weeks*. *So*, *em*, *I’d been left in limbo a little bit till I went to fracture clinic and then*, *eh*, *I went in there*.”

Participant 12-A

However, this was not the case for all participants.

“*And then they referred me to the fracture clinic the day after*. *It was excellent*. *No waiting*, *straight in*. *Seen*, *em*, *x-rayed again and then out again*.”

Participant 5-A

Some participants reported that they would have benefitted from more information about the process of navigating the healthcare system.

“*On the first night*, *when I went to hospital*, *a little bit more information*, *something written down would have*, *I was told you’ll be contacted*, *and you’ll have an appointment with a consultant so possibly just sort of a bit more information at that time*”

Participant 1-A

Some participants reported that they would have liked more information on what they should or should not be doing while their injury is healing. They felt that they were left to figure out what to do themselves.

“*When they did say we think you fractured your shoulder*, *put my collar and cuff on and sent me on my way*, *it was only afterwards that I thought*, *well*, *what part of my shoulder*, *you know what I mean*, *what do I need to do?*”

Participant 13-A

#### Guidance

Some aspects of the clinical consultation led to participants feeling reassured, in that they were progressing as expected. Participants appreciated the guidance from clinicians regarding what they could do and what they should avoid.

“*He’s been encouraging me*, *he’s told me that I’ve not done anything wrong*, *telling me what I’m doing right*. *He’s very pleased with the range of movement that I’ve got back in my arm*. *He’s been really encouraging and he’s quite relaxed as I say*, *which helps you feel more relaxed as well*.”

Participant 1-A

“*I did think the physio there was really good*, *I felt a lot better when I came out because he did explain a lot of the things*, *so I felt quite bucked up when I came out after seeing him*.”

Participant 8-A

#### Change in information needs over time

Information needs changed over time, with information about the extent of the injury and practical advice needed first. Reassurance and advice on how to progress their rehabilitation were important to people later in their recovery (by the time of the second interview).

*“I was told where the fracture was*. *It would have been nice for me to just have you know to have an x-ray or scan or whatever shown to me so it could be pointed out so I could see it with my own eyes like you know what I mean?”*

Participant 10-A

*“And every time I’ve had an appointment*, *he’s told me you’re exactly where you should be*, *you know*, *you’re doing everything right because I’ve had to show him the exercises of course that we’ve been going through”*

Participant 1-B

### Theme 4:”*Getting back to*, *you know*, *as I were before my accident”*: Recovery after a shoulder fracture

#### Achieving recovery

Recovery meant different things to different people, some wanting to be completely pain-free and just like before their injury.

“*Well*, *getting back to full fitness and getting back to*, *you know*, *as I were before my accident*. *That’s my ultimate goal… I want to be able to get to a position where*, *you know*, *it were as if nothing had happened*.”

Participant 12-B

However, most people were concerned about regaining the function in their arm to be independent.

“*What’s more important so long as I still have the functionality of my arm and shoulder*, *then you know*, *at my age I’m not really concerned about it being beautiful*, *you know what I mean? [laughing]*”

Participant 10-A

“*My independence is the most important to me*.”

Participant 11-A

There were inconsistencies in what people were told with regards to timelines to recovery.

“*I’ve been told that it could take 6 to 8 weeks*, *now when I went to physio*, *he’s put it up to months*, *like it could be 12 months*. *It all depends on*, *like your age*, *and how you manage things normally*.”

Participant 16-A

Many felt they had made a good recovery by the second interview at 4.5 to 6 months after the injury, although the recovery was not complete at this stage. Some commented on how slow the recovery was.

“*I have to say in the last few months it has got a little bit easier*. *It’s still not 100%*, *still struggling with certain tasks and particularly in the strength side of things*, *the strength and just feel really weak with this shoulder now*.”

Participant 13-B

“*I think it’s not recovering well but it is recovering*, *but it’s slow*.”

Participant 9-A

Some felt they would never fully recover.

“*It’ll feel different for the rest of my life*, *you know*.”

Participant 6-A

#### Factors that influence recovery

Many felt that exercise was a valuable part of their recovery, and this was enabling them to be active participants in their rehabilitation, giving them some power and control over their recovery.

“*Keep doing your exercises*. *I think that is so important*. *Without that it’s going to stiffen up and you won’t be able to*, *you know*, *get back to where you were really*. *I just need to match it up with my other side so that’s how I know whether it’s progressing*.”

Participant 5-B

“*I think it’s up to me*, *em*, *to do the exercises*. *I got a lot of input*, *it’s up to me to keep doing my exercises*.”

Participant 14-B

Appropriate communication in the form of reassurance was also found to be a contributing factor to recovery. One participant, when asked if he was in much pain, stated that the pain had eased because he had been given some reassurance about the healing in the bone.

“*[I have] only a little bit [of pain] because I got a letter from the consultant fellow*, *the fracture clinic and all that and he confirmed that yeh*, *everything is*, *in fact he let me see the x-ray*, *to see how it had healed up*, *kind of thing*, *you know*.”

Participant 10-B

Determination was thought to be a factor that influenced recovery for some participants.

“*It was much quicker than I thought it would have been actually*, *you know*, *I seem to have got back to normal a lot faster than I thought I would do*. *According to my daughter*, *it’s because I was determined but whether it was or not…*”

Participant 8-B

Gradual recovery resulted in a gradual improvement in control.

“*I feel OK now that I’m back at work and I’m on a phased return*. *So*, *although initially I felt a little bit powerless*, *now I do feel that yes*, *I am in control*.”

Participant 13-B

Some participants reported that they were not in control of their injury or their recovery and that it was up to a higher power.

“*Praise be to God*, *he’s the only one that can control it*, *do anything about this world really*.”

Participant 9-A

“*It was an accident*, *it was God’s decision that this had to happen*, *so the accident took place*.”

Participant 2-A (via interpreter)

#### Change in recovery over time

Many participants were initially concerned with movement in the arm, but their priorities changed to function over time.

*“[Recovery] is to try to get back to be able to move the arm you know.”*

Participant 8-A

*“I haven’t been back to physio cause he said there wasn’t a need for it*, *just to carry on the exercises at home and I’ve gone back to Zumba classes and so I can do most things*.*”*

Participant 8-B

*“I were more concerned about*, *you know*, *what I’d actually done and whether I’d actually get*, *you know what*… *you know*, *the final outcome*, *would I get full movement back in my arm basically*.*"*

Participant 12-A

*“Well*, *getting back to full fitness and getting back to*, *you know*, *as I were before my accident*. *That’s my ultimate goal*. *I want to be able to get to a position where*, *you know*, *it were as if nothing had happened*.*”*

Participant 12-B

## Discussion

This study explored the patient perspective of living with a shoulder fracture, and to understand what is important to these people with regards to information provision and recovery. This is important to be able to provide the right information for patients at the right time, and in the right way.

Four themes were identified: dependence, vulnerability, information needs and recovery. Dependence, or lack of independence was mentioned by all participants. This is consistent with previous qualitative studies with patients after a fracture [8,23]. A qualitative systematic review of patient perspectives after hip fracture reported that physical functioning, mobility and independence are important factors in recovery [24]. Participants in the current study reported feeling that they were being a burden, which was also reported by King and Hebron [25], in patients with frozen shoulder. However, this loss of independence goes further than merely not being able to care for oneself. There was a sense of loss of identity reported by some participants in this study, due to changing roles. The role reversal from care giver to cared-for person and the loss of role as a worker (when needing to take significant amounts of time off work due to injury) significantly affected those who described these experiences. Genneralli *et al*. [26] report that sports people commonly suffer from a loss of identity after a musculoskeletal injury. Loss of identity following musculoskeletal injury is also reported by Saunders *et al*. [27], in relation to their job. Stern *et al*. [28] report that emotional aspects of recovery are important to patients after a distal radius fracture, and that these should be addressed by clinicians, along with expected timeframes to recovery, to minimise loss of control due to uncertainty. This loss of control was also evident in this study of shoulder fracture patients, especially in the initial stages (first interview). The feeling of control was improved in most participants by the time of the second interview, which tended to align with improved communication and guidance from health professionals, though other factors may also influence participants’ feelings of control.

There is evidence that rehabilitation priorities change with time in major musculoskeletal trauma [13] and this has been shown to be the case for those with shoulder fractures in this study. Shoulder function and independence in self-care develop as priorities in the early stages after a shoulder fracture, and later, returning to other meaningful activities such as work and hobbies. The recovery process is complex and there are various factors that influence recovery. It has been suggested that pain, function/disability and recovery/healing can be thought of as recovery outcomes in themselves but also, they are factors in overall recovery [29]. Vulnerability, including fear of falling and feelings of frailty were common among participants in this study. While some participants reported that their fear of falling reduced as time passed, many had persisting feelings of vulnerability at six months. Vulnerability combined with reduced function led to feelings of isolation, where participants reported being unable to go out like they previously did. After a fracture, patients may have a lack of active engagement in their rehabilitation activities due to feelings of vulnerability [30].

Communication is an important factor in healthcare and can negatively or positively affect patient experiences and outcomes [31]. Appropriate and adequate information provision is an essential part of communication within a therapeutic encounter, and both information provision and communication have been identified as important factors in patient-clinician interactions [32]. As can be seen from the quotes in this study, communication and information can have either positive or negative influences on the patient experience and their recovery. The lack of adequate communication that participants reported, led to a sense of abandonment, with long wait times and not knowing what they could do to help themselves. However, good clear communication, was reported as being helpful in some cases, in that participants felt reassured and had guidance on how they could actively participate in their recovery, for example actively participating in rehabilitation activities. Clear communication and information with shorter waiting times were aspects that have been identified previously as being important in recovery from injury [11,24]. Information needs changed over time, with information about the extent of the injury and practical advice needed first, but later participants emphasized information about reassurance and recovery. This is consistent with previous research reporting that information needs of major trauma patients [33] and ankle fracture patients [9] change over time as recovery progresses. Moos *et al*., [11] also reported that some participants felt the benefit of guidance by healthcare professionals through their recovery. The theme of information needs was strongly linked to most other themes found in this study. This is similar to what was found by Moos *et al*., [11] in their study of patient’s experiences after a distal radius fracture.

Those participants who reported religious or spiritual reasons for their injury showed acceptance of their injury. Similar findings have previously been reported [34] where those with spiritual or religious beliefs were accepting of their injury and using positive reframing of their injury. Wiese-Bjornstal et al. [34] also reported that those with religious or spiritual beliefs were less likely to feel that they had direct responsibility for their recovery from sports injury. This may affect the recovery of participants from shoulder injuries in a similar way. It has been shown that those with stronger spiritual or religious beliefs cope better with injury than those without [35].

As demonstrated by the participant quotes from this study, after a shoulder fracture, recovery goes beyond regaining range of movement and function. Feelings of vulnerability and isolation are important factors to consider after a shoulder fracture as these significantly affect daily life. Clinicians should be aware of the full impact of these injuries to effectively guide patients to a full recovery, including overcoming feelings of vulnerability and regaining their sense of identity. Adapting communication appropriately to the changing needs of the patient along the timeline to recovery may help the recovery process.

### Study strengths and limitations

An attempt was made to recruit a diverse population regarding age, gender, levels of deprivation (based on IMD) and ethnicity. The participants that were recruited were predominantly white female, which corresponds with previous literature [36,37] stating that white females have a higher fracture risk than males or those from other ethnicities. However, the extent to which these findings can be applied to the non-white female population is open to question.

Three participants declined a follow-up interview, therefore their views on recovery were limited to the first few weeks after injury. This was a single centre study, only recruiting those patients who were referred to outpatient physiotherapy, so the results may not be transferable. However, the study participants were purposively recruited to represent the population who sustain a shoulder fracture.

The lead author, who carried out the interviews, is a physiotherapist trained in qualitative methods. Although the lead author was not known to the participants, she introduced herself as a physiotherapist-researcher, which may have impacted on the relationship between the interviewer and participant. Although also a physiotherapist, the interviewer analysed the interviews through the lens of a researcher, focusing on the participants’ viewpoint, the use of numerous quotations in the write-up place the focus on the participants viewpoints.

The interviews were conducted by telephone which may have affected the rapport between the interviewer and participant, in that response to non-verbal queues was impossible. This may affect the depth of findings. However, from the data, an adequate depth of responses was generated due to the semi-structured interviews, which started off with an introduction and a very general question to build rapport: ‘Could you begin by telling me about your experience of your shoulder injury?’ The use of prompts such as ‘Could you tell me more about that?’ helped to gain a deeper understanding of participants responses. The length of the interviews may have been a limit to the depth of findings, but participants were given time to think and respond to questions. The follow-up interview was a second opportunity for participants to include all the relevant aspects related to the study.

Transcripts were not sent back to participants for checking, partly due to the longitudinal nature of the study, which explored participants views over time. Views may have changed further by the time the transcripts were sent for review. Also, the value of participant transcript checking is debatable in the literature [38,39]. Instead, an overview of the findings was discussed with the PPI group, which formed part of the critical review and refining of the themes.

## Conclusions

The path from injury to recovery following a shoulder fracture is multi-dimensional, including loss of function and independence, vulnerability, change in identity and isolation, all of which are important to the person who sustained the injury. It is important to ensure adequate communication and information provision to enable people to have the knowledge and control to be active participants in their recovery. Adopting a person-centred care approach, taking into account the changing priorities and information needs of patients throughout their recovery journey may lead to improved patient care. Future research is needed to design information resources that meet the needs of patients along their timeline of recovery.

## Supporting information

S1 File COREQ checklist. (DOCX)

S2 File Flow chart of programme of work. (DOCX)

S3 File Interview topic guide. (DOCX)
