# Supplementary material for: Exploring the patient’s recovery journey and information needs following a shoulder fracture: A qualitative interview study
Source: PLoS One. 2024 Dec 31;19(12):e0316516. doi: 10.1371/journal.pone.0316516 (PMC11687763; doi:10.1371/journal.pone.0316516)
Supplement: S2 File — (DOCX) [file pone.0316516.s002.docx]

**S2:**

**Flow chart of programme of work**

This study – qualitative interview study exploring the patient’s perspective of living with a shoulder fracture and recovery and information needs.

Co-design and development of appropriate information resources for these patients who have sustained a shoulder fracture.

Analysis of publicly available information sheets for shoulder fractures – to understand the readability and fitness for purpose.

Survey of patient information sheets (publicly facing information) on shoulder fractures – to understand current NHS practice for rehabilitation following a shoulder fracture.
