# Supplementary material for: Exploring the patient’s recovery journey and information needs following a shoulder fracture: A qualitative interview study
Source: PLoS One. 2024 Dec 31;19(12):e0316516. doi: 10.1371/journal.pone.0316516 (PMC11687763; doi:10.1371/journal.pone.0316516)
Supplement: S3 File — (DOCX) [file pone.0316516.s003.docx]

**Interview Topic Guide**

| **Title of Project:** | Living with a shoulder fracture – an interview study |
| --- | --- |
| **IRAS ID:** |  |
| **Study Number:** | DEV033 |
| **Name of Researcher:** | Pauline May |
| **Version Number & Date** | Version 1.0  Date 09/03/2023 |

**Introduction:**

- Introduction to researcher and study topic
- Explanation of the aim of the study
- Explain confidentiality and anonymity
- Explain recording length (up to 30 minutes) and nature of discussion
- Go through consent issues and explain they may withdraw at any time, and they do not have to answer any questions they would prefer not to
- Check whether the participant has any questions
- Check the participant is happy to continue

**Demographics**

Check consent, age, post code, time since injury, dominant or non-dominant arm.

**Start recording**

**Initial experience of the injury**

Can you begin by telling me about your experience of your shoulder injury?

Prompts: Initial incident, diagnosis, treatment

How did you find your experience in Urgent Care Centre/fracture clinic/physiotherapy?

Prompts: Interactions with staff, information provided, terminology used, treatment options

How did you feel in the days and weeks following the shoulder injury?

Prompts: Physical feelings as well as emotional/mental, coping strategies

**Effects on self, family, life**

How did your shoulder injury affect those around you, family, friends, neighbours, colleagues etc.?

Prompts: help from others, changing plans

Thinking about your daily life, what are your problems since your shoulder injury?

Prompts: washing, dressing, hobbies, driving, walking

Thinking about yourself, what is important about you and the way you handle your shoulder injury?

Prompts: coping strategies, mental outlook

Could you tell me about any surprising effects of the shoulder injury?

Prompts: Anything unexpected

Do you feel like you are coping with your shoulder problem overall?

Is there anything you avoid because of your shoulder problem?

What do you think about how your shoulder is at the moment?

What do you think about how your shoulder will be in the future?

Was there anything that made you cross or frustrated along the way?

Is there anything you figured out for yourself that was useful?

**Information**

What key advice were you given?

Prompts: Immobilisation, pain relief, function

How did you feel about this advice?

Prompts: Adherence/deviation from advice

Thinking about your recovery, what do you know now that you would have liked to have known sooner?

Prompts: Information that may be beneficial, timelines to recovery, expectations

**Conclusion**

Is there anything further you would like to mention or discuss?

Summarise and check key issues?

Thank the participant for their time

Advise on telephone call to arrange second interview

**Additional probing questions**

How did/does that make you feel?

Could you tell me more about that?

Can you please give me an example?

Why do you think that is?
